# Treatment Comparison for Medial Femoral Condyle Subchondral Cystic Lesions and Prognosis in Yearling Thoroughbred Racehorse Prospects

**DOI:** 10.3390/ani14071122

**Published:** 2024-04-06

**Authors:** Marcos Pérez-Nogués, Gabriel Manso-Díaz, Michael Spirito, Javier López-Sanromán

**Affiliations:** 1 Peterson & Smith Equine Hospital, 4747 SW 60Th St., Ocala, FL 34474, USA; mperez.vet@hotmail.com; 2 Department of Animal Medicine and Surgery, Universidad Complutense de Madrid, Avenida Puerta de Hierro s/n, 28040 Madrid, Spain; lsroman@ucm.es; 3 Hagyard Equine Medical Institute, 4250 Iron Works Pike, Lexington, KY 40511, USA; mspirito@hagyard.com

**Keywords:** horse, osteochondrosis, subchondral cystic lesion

## Abstract

**Simple Summary:**

Subchondral cystic lesions (SCL) are a common finding in young Thoroughbreds intended for racing. Although the complete etiology is not fully understood, it is believed that SCL is a form of osteochondrosis that can also be induced by trauma. Several treatments have been studied to improve soundness or athletic prognosis. The objectives of this study were to compare the athletic prognosis of four current treatments performed in Thoroughbred yearlings, to compare auction value and athletic ability with maternal siblings without medial femoral condyle SCL, and to detect if the size of the SCL would have a negative effect on the racing prognosis. The treatments studied were intralesional injection of corticosteroids, SCL debridement through the joint with a drill bit, translesional cortical screw placement, and absorbable hydroxyapatite implant placement. The price paid at an auction for horses treated for SCL was significantly lower than that of their siblings. Horses treated for SCL had significantly lower chances to start in a race than their siblings free of pathology (59% and 74%, respectively). Wider SCL negatively affected the chances of starting in at least one race and the earnings as a 2-year-old racehorse. Horses with SCL treated with a bioabsorbable implant had a higher median in starts as 3-year-olds than horses treated with debridement of the SCL with a drill bit. In conclusion, Thoroughbred yearlings treated for a medial femoral condyle SCL had lower auction prices and decreased ability to start a race compared to siblings; wider cysts had worse prognosis to start a race and might affect earnings as 2-year-olds; and horses treated with bioabsorbable composite implant placement had more starts as 3-year-olds than other techniques.

**Abstract:**

Subchondral cystic lesions (SCL) in the medial femoral condyle are a usual finding in Thoroughbred survey and auction repository radiographs. Several treatments with different outcomes have been studied over the years to improve soundness and racing prognosis. Our objective was to report the racing prognosis in Thoroughbred yearlings intended for racing that were diagnosed with SCL in the medial femoral condyle and were treated using four current and different techniques: intralesional injection of corticosteroids, SCL debridement through the joint with a drill bit, translesional cortical screw placement, and absorbable hydroxyapatite implant placement. Data from 182 Thoroughbred yearlings treated for SCL in the medial femoral condyle were collected from 2014 to 2020. Limb affected, age at surgery, sex, and radiographic measurements of the SCL were recorded. Auction price and racing performance were collected for treated horses and compared to 154 maternal siblings free of medial femoral condyle SCL. Analyses were conducted to assess if racing prognosis was affected by SCL size, to detect differences in auction price and selected flat racing outcome parameters between cases and controls, and to compare racing prognosis between the studied treatments. Mares and lesions located in the right stifle were significantly overrepresented. The auction price of treated horses was significantly lower than that of their siblings. Horses treated for SCL had significantly lower chances to start in a race than controls (59% vs. 74% respectively). Wider SCL negatively affected the chances to start at least in one race, and negatively affected the earnings made in the 2-year-olds’ racing year. Horses with SCL treated using a bioabsorbable implant had a significantly higher median in starts as 3-year-olds (seven starts) than horses that had the SCL debrided with a drill bit (three starts). In conclusion, Thoroughbred yearlings treated for a medial femoral condyle SCL had lower auction prices and decreased ability to start a race compared to siblings’ wider cysts had worse prognosis to start a race and might affect earnings as 2-year-olds; and horses treated with bioabsorbable composite implant placement had more starts as 3-year-olds than with other techniques.

## 1. Introduction

Subchondral cystic lesions (SCL) have a predilection for the medial femoral condyle, with a prevalence of 0.08–9.65% in Thoroughbred yearling repository radiographs, and account for 45.8% of SCL in all locations [1,2,3]. The etiology of SCL is not completely understood; however, trauma or osteochondrosis are likely implicated [4,5,6,7]. Most SCL develop at an early age, and clinical lameness is not always detected in lameness evaluations [1,8]. Radiographic monitoring of SCL at a yearling age is a common practice to determine if a surgical or medical intervention should be recommended [1,8,9].

Treatments for SCL have been extensively studied [10]. Current treatments for medial femoral condyle SCL include rest, intra-articular or SCL intralesional injection with corticosteroids, plasma-derived products or stem cells, arthroscopic or external debridement with or without filling of the defect with cancellous bone grafts, cartilage grafts, mosaic arthroplasty, and stainless-steel screws or composite absorbable transcondylar implants [4,9,11,12,13,14,15,16,17,18,19,20,21,22,23,24]. The racing prognosis of Thoroughbred yearlings and racehorses treated for SCL in the medial femoral condyle is 64–84% depending on the technique used [9,19,20,23], and the subjective soundness prognosis is 56–95% among different studies [11,12,13,14,15,18,20,23]. SCL show marked variation in size and shape, which can change over time radiographically [1,25]. They are usually surrounded by mild to marked subchondral bone sclerosis, and complete radiographic resolution after treatment is rare and not always correlated with soundness or racing prognosis [1,9,10].

When evaluating racing prognosis after arthroscopic debridement of SCL, more than 15 mm of cartilage surface disruption was found to be associated with poor racing prognosis [19]. Shallower lucencies have been found to have a better racing prognosis than SCL [1], and deeper SCL located in distal extremity bones were associated with fewer wins and placed races [26]. Different shape or morphological classifications have been made, but they failed to detect any impact in prognosis despite theoretical differences in subchondral bone mechanics [6,26,27]. Other parameters that have been associated with lower response to treatment were age (horses > 3 years old), the presence of bilateral stifle SCL, and concurrent stifle pathology [5,21,28].

The objective of this study was to compare the athletic prognosis of Thoroughbred racing prospect yearlings that underwent four different treatments for SCL in the medial femoral condyle, and to compare their performances as racehorses and their auction price to maternal siblings. A secondary objective was to detect if SCL size could affect racing prognosis. The treatments included in the study were ultrasound-guided intralesional corticosteroid injection, transarticular debridement using a drill bit, translesional cortical screw, and bioabsorbable implant placement. We hypothesized that yearlings with radiographic evidence of SCL would have a lower auction price at a yearling age, worse racing statistics in terms of earnings and won and placed races, and shorter athletic careers with fewer starts. We also hypothesized that there would be no difference in racing outcomes among treatment groups, and that SCL size would have an effect on racing quality.

## 2. Materials and Methods

### 2.1. Data Collection

Data were collected from Thoroughbred yearlings (under 24 months of age) that underwent treatment for a SCL in the medial femoral condyle from 2014–2020 at a referral institution in Kentucky, USA. Data collected included age in days at the time of the procedure, sex, limb affected, type of treatment, and lesion size. Auction purchase price as a yearling age and racing data including total number of starts, wins, placed races, number of starts as 2- and 3-year-old, earnings as 2- and 3-year-old, total earnings in US dollars of horses that were determined to be retired from racing, and age in days at the time of the horse’s first race were collected from a public database (equibase.com) with the date of 1 November 2023. A control group with maternal siblings that were born a year prior or a year later without a clinical history of SCL in the medial femoral condyle was selected for statistical comparison. Retirement from racing was determined by absence of any recorded race or timed training within 60 days prior to data collection. This time was chosen since most horses actively competing in this study had recorded training or racing records at least biweekly until they abruptly stopped, and the longest period of inactivity between recorded events was 56 days in this study population.

### 2.2. Image Analysis

**Figure 1:**
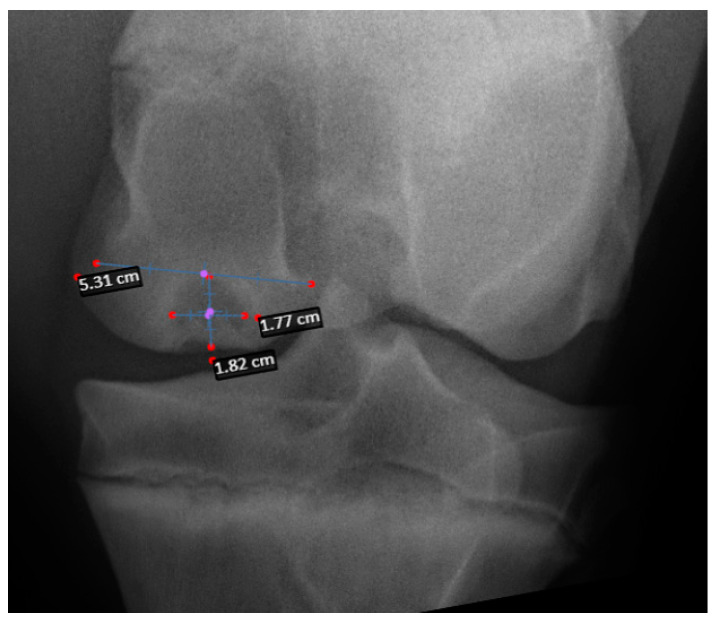
Craniocaudal radiograph of the stifle in a horse with a medial femoral condyle SCL. Measurements taken to calculate the index of the measured SCL with the total width of the medial femoral condyle affected to diminish magnification measurements errors.

Pre-operative radiographs in DICOM format were evaluated using a medical imaging viewer software (SmartPacs 2.0, Sound, Carlsbad, CA, USA). The width at the joint surface and height of the lesion was measured in the standard stifle craniocaudal radiographic view (caudo10˚proximal-craniodistal oblique) (Figure 1). Magnification was corrected by calculating the index of the measured SCL with the total width of the medial femoral condyle affected.

### 2.3. Treatments

Horses were classified into four groups based on the type of procedure performed. Treatment groups were ultrasound guided SCL injection with corticosteroids, transarticular SCL debridement, cortical screw placement, and absorbable hydroxyapatite composite implant placement. All treatments were performed before the horses were presented for sale in an auction at yearling age (12–23 months of age).

A corticosteroid injection was administered under general anesthesia with the horse placed in dorsal recumbency with the affected stifle flexed. The site of the injection was determined with ultrasound and, after clipping and sterile preparation of the area, the SCL was injected with 80 mg of methylprednisolone acetate in 70 cases (Depo-medrol 20 mg/mL, Zoetis, Kalamazoo, MI, USA), 12 mg of betamethasone acetate in 12 cases (BetaVet 6 mg/mL, American Regent, Inc. Animal Health, Shirley, NY, USA), or 10 mg of triamcinolone acetonide in 13 cases (Kenolog-10, Bristol-Myers Squibb, Princeton, NJ, USA) at several spots in the SCL lining using a 18 G by 3.5′ needle. After the corticosteroid injection, 3 mL of a glycosaminoglycan suspension (Polyglycan 10 mL vial, Bimeda, Oakbrook Terrace, IL, USA) mixed with 125 mg of amikacin (Amikacin Sulfate Injection 500 mg/2 mL, Avet Pharma, East Brunswick, NJ, USA) were injected into the affected medial femorotibial joint. This intralesional injection technique was similar to the one used in SCL in the distal extremity [26]. To perform SCL debridement, the patient was positioned in dorsal recumbency with the leg flexed. A 3.2 mm-sized drill was positioned under radiographic guidance through the defect in the condyle perpendicular to the joint surface and through the medial femorotibial joint. Three to four divergent holes towards the subchondral bone in a disto-proximal direction were made. Cortical screws and absorbable implants were placed as previously described [20,21,26]. In brief, the horse was anesthetized and placed in dorsal recumbency with the leg extended. The cortices were drilled with the correct drill bit size for the implant selected. The drilling direction was guided radiographically across the bone in a slight proximal to distal angle travelling through the center or proximal third of the SCL. All cortical screws were self-tapping 4.5 mm placed in lag fashion, whereas all absorbable implants were 20 mm long by 9 mm wide placed into the SCL placed in a neutral position.

### 2.4. Statistical and Data Analysis

Data were analyzed with SPSS statistics version 29 (IBM, Armonk, NY, USA). All numerical data did not have a normal distribution using a Shapiro–Wilk test. A Wilcoxon rank sum test comparing cases and controls was performed to detect differences in sale value, total career earnings in already retired horses, earnings as a 2- and 3-year-old, total number of starts, number of starts as a 2- and 3-year-old, and wins, places, and age at first race. A Pearson Chi square test was used to detect any differences between the leg affected and the chances of the horse starting a race, to detect differences between the sexes in horses with SCL in the ability to start at least one race, to compare the sex distribution between cases and controls, and to compare the ability to start a race between cases and controls. A Kruskall–Wallis equality-of-population test was performed to detect differences between the four treatment groups for the parameters of sex, auction price, ability to start a race, total earnings, earnings as a 2- and 3-year-old, total number of starts, starts as a 2- and 3-year-old, age at the first race, age at surgery, number of wins, number of placed races, width, and height of the SCL. A Mann–Whitney U test was used for pairwise comparison in the significantly different parameters detected. A multivariate negative binomial regression analysis was conducted to determine if sex, age at surgery, SCL height, SCL width, type of surgery, and leg affected could predict the total number of starts, number of starts as a 2- and 3-year-old, number of wins, and number of placed races, total career earnings, and earnings as a 2- and 3-year-old. A binary logistic regression was conducted to determine if height, width could predict the ability of a patient to start in at least one race. For all tests and regression model fitting, a *p*-value ≤ 0.05 was considered significant.

## 3. Results

During the study period, 182 horses were treated for SCL in the stifle joint. Of those, 82 (45%) were sold in a yearling public auction, 107 (59%) started at least in one race, 58 (32%) raced as a 2-year-old, and 96 (53%) raced as a 3-year-old. The control group consisted of 154 maternal siblings, from which 94 (61%) were sold in a public auction, 114 (74%) started in at least one race, 73 (47%) raced as a 2-year-old, and 110 (71%) raced as a 3-year-old. The ability to start a race was significantly greater in the controls than in horses with SCL (*p* < 0.01). Data about racing parameters in horses with SCL and controls are compiled in Table 1. The auction price paid at yearling age was significantly higher in controls than in horses with SCL (*p* < 0.01).

Sex distribution was equal in the control group with 79 (51%) males vs. 73 (49%) females, whereas females were more prevalent in the SCL group (119 fillies; 65%) than males (51 colts; 35%). This sex distribution difference was statistically significant (*p* < 0.01). There was no difference in the ability to start a race between males and females treated for SCL (*p* = 0.47).

The SCL was located in the right stifle in 127 horses (70%), in the left stifle in 43 horses (24%), and in both stifles in 12 cases (6%). There was no difference between the leg affected in the ability to start a race (*p* = 0.75). There was no difference in the ability to start a race between unilateral and bilateral affected horses (*p* = 0.78). The median age at surgery in the affected horses was 414 days. Horses were operated on at a similar age among the different surgeries (*p* = 0.64). Greater height was associated with higher width (*p* = 0.01), and both were significantly different among treatment groups (*p* = 0.01). Horses with intralesional injections with corticosteroids had the lowest SCL width and height median calculated index, measuring 30% and 31% of the total medial femoral condyle width, respectively. The corticosteroid intralesional injection group had a significantly smaller width and height index than SCL of horses treated with translesional cortical screws (36% width and 42% height index; *p* < 0.01) or absorbable interference implants (32% width and 37% height index; *p* = 0.01 and *p* = 0.05 respectively). No difference was found in the SCL width and height index between horses treated with drill debridement and any of the other treatments (29% width and 35% height). Regression analyses showed that width but not height was found to be a significant predictor variable of the ability to start a race (OR = 0.948 [95% C.I. = 0.889–0.997]; *p* = 0.04) and the amount of earnings as a 2-year-old (IRR = 0.913 [95% C.I. = 0.865–0.963]; *p* < 0.01). For a 1% increase in SCL width the model predicted a 4.8% lower chance of starting in at least one race and 8.7% chance of lower earnings as 2-year-old.

Data comparing the athletic prognosis for the different types of surgeries can be found in Table 2. The number of starts as a 3-year-old was the only parameter with significant differences between treatment groups and was significantly lower in the transarticular drilling debridement group compared to the interference absorbable screw placement group (*p* = 0.02). None of the parameters studied (age at the time of surgery, sex, SCL height and width, and auction price) showed an effect on the ability to start a race, the total earnings, earnings as a 2- and 3-year-old, total number of starts, starts as a 2- and 3-year-old, number of wins, number of placed raced, nor age at the first race.

## 4. Discussion

The two primary objectives of the study were to assess the racing prognosis of Thoroughbred yearlings treated for SCL in the medial femoral condyle compared to maternal siblings, and to compare four different treatments. As hypothesized, we found that yearlings without SCL in the stifle joint had a better chance of starting in at least one race and had higher auction prices when sold at public auctions. However, we found no differences between SCL-treated horses and controls in their performance once they started racing. When comparing the four treatments studied, we found that horses treated with bioabsorbable implants had the highest number of starts as 3-year-olds, whereas those treated with transarticular debridement had the lowest.

Auction price in treated horses was significantly lower than controls. This was expected and in agreement with the literature [1,26]. Treatments were not randomly assigned. This is a limitation of the study, and it was confirmed that horses with wider and higher SCL were more likely to be treated with cortical screws, and horses with shallower and narrower ones were more likely to be treated with corticosteroid injections. It is likely that surgeons and owners/farm managers selected different treatments based on their experience, but we found that the radiographic size of the SCL affected their treatment choice. The ability to accurately place an implant within the SCL might be subjectively easier in larger SCL and could favor the decision to choose these techniques. Higher and wider cysts also allow placement of the implant at a higher distance from the joint surface, which is likely to reduce complications due to the implant, screw, or drill bit contacting the medial femoral condyle articular surface. A study evaluating similar techniques also found that bigger SCL were more likely to be treated with cortical screw placement rather than with corticosteroid injections [21]. This was probably caused by a false perception of an increased effectiveness from owners and referring veterinarians, as cortical screw placement is often used when other treatments have failed.

Wider SCL were found to be associated with lower ability to start a race and with lower 2-year-old earnings. One study found that a wider defect at the joint surface measured arthroscopically was the main reason for decreased racing prognosis [19]. The radiographic width of the SCL at its widest point might be correlated with the size of articular surface defect, resulting in the same effect in prognosis. We standardized the measurement of the total width of the medial femoral condyle to mitigate radiographic magnification and to better compare the articular effect in horses of different sizes. In concordance with other studies with radiographic measures of SCL in the medial femoral condyle, we failed to detect any effect of SCL height and prognosis [9,21]. However radiographically wider, and higher SCL located in the distal limb (metacarpus/tarsus and proximal and middle phalanx) are reported to have detrimental effects on the number of wins and placed races compared to siblings [26].

The predominance of right stifle SCL over left stifle cysts aligns with previous literature [1,2,9,23]. Contrary to one study, bilaterally affected cases were not found to have a worse prognosis to start a race [28]. However, more bilateral cases would be needed to confidently compare unilateral and bilateral cases in this and other studies [4]. No concurrent pathology was found in radiographs of the stifle joint of any of the horses in this study. The shape of the SCL, the evidence of sclerosis of the surrounding subchondral bone, and the radiographic resolution of the pathology were not studied.

Debridement using a drill by accessing the SCL through the joint instead of extra-articularly from the side has not been reported. This technique was designed to allow communication between the joint surface and the more vascularized and cellular spongious bone in the proximal medial femoral condyle and even extending to the marrow in metaphysis of the femur. The prognosis to start a race found with this technique (67%) was similar to the prognosis reported after arthroscopic debridement (72%) [9,18]. When arthroscopic debridement was used to treat SCL in a recent study, they found an initial enlargement of the lesion the first 2–3 months in all cases before improving by 6 months [9]. Contrary to arthroscopic debridement, expansion of the lesion could not be seen with the drilling technique, but anecdotally the drill tracts could be noticed proximally to the lesion in subsequent follow-up radiographs in an unknown number of cases that had follow up radiographs.

Computer models have been used to analyze the theorical benefits of transcondylar screw placement in the transfer of vector loads and potential bone formation stimulus inside the SCL [27,29,30]. Based on these models, bone stimulus was theorized to be increased when placing the screw in a proximal to distal oblique orientation and in lag fashion when compared to a horizontal orientation [27,29]. Also, bicortical screw engagement through the SCL was shown to be superior to unicortical engagement in retaining compression across a cyst analog [29,30]. Thus, in this study all screws were placed bicortically, in a proximal to distal oblique orientation, and in lag fashion. However, screw engagement of the cyst, proximodistal positioning of the screw, or central screw placement within the cyst in vivo were not found to have any association with racing prognosis, lameness resolution, or new bone stimulation [23].

A new technique for cortical screw placement was recently published using arthroscopy to guide screw positioning though the lesion [23]. The benefits proposed for this versus the previously published technique followed were increased reliability that the screw is going through both the cis and trans sides of the SCL, live three-dimensional alignment and monitoring of the drilling direction, and a more accurate selection of screw size [21,23]. The disadvantages compared to the previously published technique that we used is the need for arthroscopic equipment, more assistants required (4 vs. 2), the need for incisions into a synovial cavity, and presumably longer intraoperative time.

We found that 56% of the horses started a race after intra-articular corticosteroid injection, similar to a recent publication on racehorses from Europe (59.7%), but lower than the 67–77% success rate previously described in the literature on racehorses from the United States [9,12,23,24]. This might be related to the use of methylprednisolone acetate in most of the cases of this study and in some of the cases of Young’s study, which resulted in a lower percentage of horses returning to soundness compared to those treated with triamcinolone acetonide [12,24]. Cortical screw placement also had a similar prognosis than previous studies looking at soundness or return to function in different breeds (60–62.5%), and had similar prognosis to start or return to race in Thoroughbred racehorses (57.8 vs. 57.7%) [21,23]. The metallic cortical screw will be present in post-operatory radiographs unless it is removed. This might be a radiographic blemish that buyers avoided and may have lowered the auction price of this group. However, it was not significant when compared to the other techniques. The racing ability of Thoroughbred yearlings treated with bioabsorbable composite implants before starting a race was higher than those reported for racehorses in Italy (58 vs. 48%) [20]. A similar prognosis was found when comparing these techniques with SCL located in the distal extremity in a population of Thoroughbred yearlings from the same region using similar treatment techniques [26]. In that study, 55%, 50% and 66% of horses started a race after SCL intralesional corticosteroid injection, cortical screw, and bioabsorbable composite implant placement, respectively [26].

Very similar results were found when comparing the median number of starts, time from surgery to first start, and median age at the first start of our study population with a population of horses in Europe in the same period after SCL treatment with similar techniques [23]. Arthroscopic debridement technique in that study was found to result in longer post-operative time to first race compared to techniques that we also used in this study.

Horses treated with debridement using a drill bit had slightly higher chance of starting a race but had worse outcomes than the other treatments in number of starts, number of placed races, total earnings, earnings as 2- and 3-year-old, and age at the first race. However, only the number of starts as a 3-year-old was found to be statistically significantly lower in the drill debridement group than the absorbable implant placement group. Regardless, we showed that all techniques had comparable results and were considered effective, but they were not good enough to re-stablish the potential that patients’ siblings had to start a flat racing career. Contrary to our hypothesis, horses treated for SCL that started racing had very similar racing results than controls despite the lower 2-year-old earnings.

There are inherent limitations as this is a retrospective study and patient data were acquired from only one location. The innate athletic ability of a horse varies among individuals, and better innate athletic ability might not be equal among treatment groups. Although sibling comparison studies are good tools to adjust for unmeasured confounding, they have inherent and inevitable limitations as there still are epigenetic, genetic, environment exposure, and time differences that can produce differences in racing prognosis between the treated individual and their sibling [31]. Both treatment and control groups might include horses with common osteochondral disease lesions that could have affected performance. Errors using the radiographic imaging viewing software measurement tool could exist as the limit of detection on the radiographic measuring tool was 1 mm. The time of 60 days of unrecorded activity chosen in this study to determine retirement for racing might not be valid for other studies as horses sustaining injuries may recover in a period longer than 60 days and return to race. Because of this time cut-off point used, there might have been some cases in which the horse returned to race later than 60 days and could affect some of the racing parameters values. However, we believe this to be unlikely based on the age of the youngest horses at time of data collection, to have a minimal impact on our data, and to not affect the parameters recorded for 2- and 3-year old racing ages.

## 5. Conclusions

Medial femoral condyle SCL negatively impacted the auction price and the ability to start a race in yearling Thoroughbred racehorse prospects when compared to siblings. SCL of greater width was associated with decreased racing performances, as defined by lower chances of starting a race and chances of lower 2-year-old earnings. Treatment of medial femoral condyle SCL with corticosteroid injection, cortical screw, or bioabsorbable implant placement had similar racing performance prognoses. Horses treated with transarticular debridement were found to have fewer starts as 3-year-olds.

## Figures and Tables

**Table 1:** Auction price and racing data results for cases and controls reported in medians (IQR). * Denotes significant difference found *p* < 0.05.

	Horses Treated for SCL	Controls
Auction Price	35,000 (10,000–80,000) *	70,000 (24,750–205,000) *
Number of horses starting a race (%)	107/182 (58.8%)	114/154 (74%)
Number of starts	13 (5–22)	11 (5–20)
Starts as a 2-year-old	1 (0–3)	1 (0–3)
Starts as a 3-year-old	5 (2–7)	5 (3–8)
Number of races won	2 (0–3)	2 (0–3)
Number of races placed	3 (1–7)	2 (1–6)
Total earnings	50,411 (14,520–120,636)	37,675 (13,945–100,751)
Earnings as a 2-year-old	8000 (1510–20,475) *	10,345 (2183–22,355) *
Earnings as a 3-year-old	15,346 (3798–40,074)	17,062 (3776–44,505)
Days of age at the first race	949 (821–1140)	950 (890–1093)
*N* (%) of horses that raced	107 (59%)	114 (74%)

**Table 2:** Performance results reported in medians (IQR) for horses grouped by type of treatment received for an SCL. * Denotes significant difference found *p* < 0.05.

	Intralesional Injection with Corticosteroids	Debridement with Drill Bit	Traslesional Cortical Screw	Composite Bioabsorbable Implant
Number of horses	95	31	20	36
Auction Price (USD)	37,000 (10,000–90,000)	46,000 (31,250–141,250)	22,000 (1000–54,492)	32,000 (11,000–60,000)
*N* (%) of horses that raced	53 (56%)	21 (67%)	12 (60%)	21 (58%)
Number of starts	13 (6–25)	11 (3–18)	13 (5–21)	13 (7–24)
Starts as a 2-year-old	1 (0–3)	1 (0–3)	2 (1–4)	2 (0–3)
Starts as a 3-year-old	5 (2–7)	3 (1–5) *	5 (1–7)	7 (3–9) *
Number of races won	2 (0–3)	1 (0–4)	2 (0–3)	1 (0–3)
Number of races placed	3 (1–7)	2 (0–4)	3 (1–7)	4 (1–7)
Total earnings (USD)	54,286 (15,080–122,345)	27,291 (1620–122,282)	38,131 (4145–115,013)	42,953 (24,407–110,886)
Earnings as a 2-year-old (USD)	10,395 (2437–26,076)	4070 (1095–70,645)	10,757 (1326–24,292)	7900 (1012–21,645)
Earnings as a 3-year-old (USD)	14,520 (3105–35,433)	4955 (1928–49,019)	13,200 (690–36,850)	17,325 (8874–40,966)
Days of age at surgery	420 (332–539)	387 (323–477)	434 (339–551)	423 (366–496)
Days of age at the first race	971 (822–1106)	1060 (854–1286)	855 (782–981)	847 (815–1084)

## Data Availability

Data are contained within the article.
